# Case of Puerperal Fever with Dropsy, Succeeded by Phlegmasia Dolens, Gastritis, and Spontaneous Hydrophobia

**Published:** 1816-01

**Authors:** James Thacher

**Affiliations:** Plymouth, Massachusetts.


*For the London Medical and Physical Journal.*

### *Case of Puerperal Fever with Dropsy, succeeded by Phlegmasia dolens, Gastritis, and spontaneous Hydrophobia.* By James Thacher, M.D. of Plymouth, Massachusetts.

AN amiable and accomplished lady, aged about twenty, a native of Denmark, having married a young gentleman of this town, performed a voyage from Copenhagen, during the first months of gestation, and arrived here in Oct. 1812. The vessel was ill calculated for the voyage; no comfortable accommodations could be afforded, and by close confinement, constant exposure to wet, and frequently to the most imminent danger, Mrs. T. was subjected to sufferings which none but the firmest constitutions could sustain, and and which required the courageous spirit of a heroine willingly to encounter. Fortunately, however, she experienced little interruption to her health, or the amusements of the polite circle, until the seventh month, when her lower extremities were severely affected with œdematous swellings, and attended with circumstances indicating depletion by the lancet. Having, in a feeble condition, advanced to the usual period, she experienced a laborious and protracted parturition, by which she was greatly exhausted, and immediately after she was seized with strong convulsions, which continued about five minutes. Every proper application was instantly resorted to, and she had no recurrence of convulsions. No unusual circumstance intervened till the fourth day after child-birth, when she was visited with coldness and shivering, severe pains over her forehead, flushings in her face, great anxiety, and restlessness. The region of the uterus and whole abdomen soon became tumefied, extremely tender and painful to the touch. To these succeeded pains in the back, hips, and sides, with laborious respiration. The lochial discharge and secretion of milk were diminished, and both were in a few days altogether suppressed; and these were followed by a total indifference about the condition and welfare of the child. The urine was turbid, and in small quantity; skin hot and dry; pulse weak, small, and about 120 in a minute; great thirst, with a dark brown tongue; prostration of strength and watchfulness. The formidable assemblage of symptoms characteristic of puerperal fever, was now completed by the accession of nausea, vomiting, and frequent discharge of dark-coloured fetid excrements. The abdominal tension and soreness were great; the pain so acute, that the weight of the bed-covering was uncomfortable, and the least motion induced sensations of the keenest distress.

The inflammatory symptoms were such as appeared to justify the employment of the lancet. I accordingly took eight ounces of blood from her arm. An emetic of ipecacuanha was next administered; and mild laxatives and enemas were employed occasionally during the whole course of the fever. Small doses of calomel and opium, and sometimes tartrite of antimony, or ipecacuanha conjoined, were my principal remedies during the first stage. Fomentations to the abdomen, and frictions with camphorated oil, and tincture of soap and opium, were ultimately applied. On the fourth and fifth days succeeding the attack, a remission of the fever occurred, and decoction of cinchona and tincture of opium were prescribed. Great distension, soreness, and pain about the abdomen, extending to the groin, with frequent frequent liquid fetid stools, still continued, as also anasarcous swellings of the lower extremities and deficiency of urine. On examination, an effusion of fluid in the cavity of the abdomen, or between the peritoneum and the abdominal muscles, was clearly ascertained. Having derived no advantage from diuretics, I resolved to try the effects of vesication, and, on the seventh and eighth days, applied two large blisters to her thighs: by her request, two others to her legs, and also one to each hypochondrium. The effects of these applications were truly remarkable: a copious discharge of limpid water issued from the vesicated parts, and a prodigious quantity of urine was evacuated, which, with the alvine discharges, continued to be involuntary for some time. The immediate consequences were, a subsidence of the anasarcous swellings, and a great abatement of the tension and soreness of the abdominal muscles and integuments. A calm and refreshing sleep ensued, and the most distressing symptoms were in a considerable degree alleviated. Still, however, the extreme debility of the system, with a small and greatly accelerated pulse, and ghastly countenance, almost forbad the hope of a favourable issue. Digitalis, and other diuretics were duly exhibited, and by the use of pulv. ipecac. et opii, a free perspiration was preserved. On the tenth and eleventh days, a cessation of the involuntary evacuations took place; the abdomen is less swollen, and is free from pain or soreness. She complains of frequent faintness, pulse small and quick: an extreme irritability of the system; and strong aversion to medicine, is manifested. She, however, receives benefit from the use of tinctura cinchonæ comp. and decoction of cinchona is directed by way of injection. Another blister is applied over the umbilical region. Doctors Hitchcock and Hayward are consulted, and the result is a persistence in the means employed, though an unfavourable termination is apprehended. Twelfth and thirteenth days she complains of increased pain and swelling of the abdomen, with diarrhœa, and diminished secretion of urine; she has flushings, heat, and accelerated pulse. Digitalis, crem-tartar, &c. are again resorted to with some effect. Fourteenth to 16th, pulse continues frequent, urine scanty, stools thin and fetid, pain and swelling considerable. No return of lochia, nor secretion of milk, yet her appetite for food and her strength have increased, and appearances in general are more favourable.

Seventeenth to 20th.—My patient is now to be cruelly afflicted with a new and unexpected complaint, *Phlegmasia dolens.* She is seized with rigours, which are followed by a lymphatic swelling, extending from her hip to her foot on one one side, attended with much pain, soreness, and irritative fever. The morbid state of the intestinal discharges still continues, and also a deficiency of urine, in despight of the constant employment of diuretic medicines. Having formerly combated phlegmasia dolens successfully by a moderate mercurial course, I was induced to prescribe, in this instance, calomel and opium in small doses, and pulv. ipecacuanh et opii occasionally, accompanied with solutions of muriate of ammonia, acetis plumbi in vinegar, as local applications to the diseased limb. Although the most distressing symptoms were soon alleviated, many days elapsed before essential amendment was observable.

Having, for a length of time, directed my assiduous application to the tedious and difficult circumstances of Mrs. T., and exhausted every resource without the desired result, it occurred to me that the *Eau Medicinale d'Husson*, cautiously administered, might have the effect of relieving her agonizing pain, and diminishing the irritable state of her system. Accordingly, on the twenty-third day from her first attack, fifty drops of the preparation described in the American New Dispensatory, second edition, as directed by Dr. Jones, were given, which produced ease, but excited some nausea and a free perspiration. Before my next morning visit, the dose had been repeated; and in four hours after she was affected with severe vomiting, which lasted about two hours: considerable bile and mucus were thrown off, and she was much exhausted. In the afternoon, an unusual garrulity, and some confusion of intellect, were perceivable. She complained of a distressing sensation of heat, and burning in her throat, and extending to her stomach, with great thirst; her pulse about a hundred. These unpleasant appearances gradually increased, and in the evening she was much discomposed, and enjoyed no sleep. From these threatening circumstances, I was not surprised to find, on the 25th day, that my miserable patient was assaulted with genuine *gastritis*. She was now exercised with intense heat in her throat and region of her stomach, extreme restlessness, anxiety, and distressing thirst, with a quick and hard pulse. She manifests a strong aversion to her best friends, whom she had been in the habit of cherishing with the most endearing affection, and is averse to all kinds of food and drink. Although assafœtida and opium were administered by way of injection, no sleep was procured, and she continued through the night in a state of wild delirium, or morose sullenness.

Twenty-sixth. The climax of miseries is now completed by the appearance of *spontaneous hydrophobia.** She labours under distressing thirst, calling most earnestly for water, and when presented, she rejects it with horror, and throws it from her with violence. A bowl of water being held near her, she paddled in it with her hands, and yet begged earnestly for water. She made every effort to avoid its approach to her mouth, and once, when her efforts were overcome, she spirted it over the bed, or in the face of her attendants, and crushed the bowl into pieces. During thirty-six hours she swallowed not a glass full of liquids; the juice which she drew from sliced oranges was her only sustenance. She became exhausted, her extremities were cold, and nature seemed to be sinking under a load of accumulated miseries. From the very irritable state of the system in general, and stomach in particular, little or no medicine could be administered, and my chief dependance was on appropriate injections; opiates thus introduced, sometimes produced a calm sleep, and afterwards some liquids were swallowed. A blister was applied to the region of her stomach, and another over her shaven head.

For a considerable time she exhibited a spectacle of horror and commiseration, by reason of a total alienation of her mental powers. Her aspect was sorrowful; now morose and sullen, and now she has a respite, and appears to suffer the keenest anguish by a consciousness of her deplorable condition. Again she relapses, and becomes outrageous, rending her clothes, and striking with her hands every object within her reach; no persuasion can sooth, no severity restrain the violence of her actions. At length, after the symptoms of hydrophobia had subsided, she was singularly affected with nervous agitation, tremor, and universal cramps and jaw-lock. She was deprived of the power of articulation; the muscles of her face, and her limbs, were contorted in a manner different from any thing of the kind which I had ever before witnessed; and these unpleasant circumstances continued with more or less severity for eight or ten days. In fact, a most distressing combination of symptoms and morbid affections continued to harass this unfortunate

* Spontaneous or symptomatic hydrophobia, in this instance, may be distinguished from rabid hydrophobia by these particulars. The patient did not manifest that remarkable degree of sensibility from the contact of external air, nor did the sight or touch of liquids excite convulsive agitations, spasms, or horror; nor was she troubled with that copious flow of viscid saliva and foaming at her mouth, which generally attend rabid hydrophobia. The leading features of both bear a striking affinity.

lady for about forty days from the period of parturition. At several stages of her successive attacks, it appeared as though life had been spun out to the last attenuated thread, or poized on a balance of the most delicate cast; but the restoring powers prevailed, and every morbid affection gradually yielded to the elastic efforts of the constitution, except that the natural periods of menstruation remain suspended.

In regard to the remedies employed, I have only to observe, that calomel and opium conjoined, proved most decidedly efficacious, by obviating the inflammatory tendency, arresting the progress of dropsical affection, correcting morbid diarrhœa, and, probably, by subduing phlegmasia dolens.

The blisters, so extensively applied, produced the most obvious and essential good effects, by abating local inflammation, relaxing spasm, and setting free an accumulated flood of water.—*New Eng. Journ.*

